# The critical need to implement pharmacogenomics in public health services: Mexico in the global picture

**DOI:** 10.3389/fpubh.2026.1748704

**Published:** 2026-03-05

**Authors:** Carlos Perezcano

**Affiliations:** 1 Center for Research in Precision Medicine and Clinical Genomics, Genomics 360, Mexico City, Mexico; 2 Universidad Contemporánea de las Américas, Morelia, Michoacán, Mexico

**Keywords:** adverse drug reactions, evidence-based policy, genomics, health equity, pharmacogenomics, precision medicine, public health policy

## Abstract

Adverse drug reactions (ADRs) are responsible for a significant proportion of hospitalizations globally, and represent a significant, yet partially preventable, burden on public health systems, particularly in resource-limited and low- and middle-income countries (LMICs), where chronic diseases and polypharmacy are highly prevalent. Pharmacogenomics (PGx), through targeted gene panels on drug-gene interactions, has shown potential to reduce ADRs, and improve prescription safety in health systems. The central hypothesis of this article is that the gradual implementation of panel-based pharmacogenomics within public health services in LMICs is possible, ethically justifiable, and potentially cost-effective, when adapted within regulatory, epidemiological, and infrastructural conditions in each region. Using Mexico as a representative case study within the global context, this manuscript synthesizes the international implementation experience and proposes a conceptual and operational framework for the gradual integration of PGx in Public Health Institutions. Estimated hypothetical projections of the clinical and economic potential impact discussed in this article—such as the potential reduction in preventable ADR-related hospitalizations and associated costs—are illustrative and inferential, based on international evidence, and not as empirically demonstrated results within the Mexican health system. The proposed model puts emphasis on scalability, regulatory framework, ethical oversight, and equity-oriented implementation as its core principles. Through the development of a hypothetical policy-relevant model, this article seeks to serve as a basis for the implementation of future pilot programs, empirical evaluations and evidence-based decisions regarding the integration of pharmacogenomics into public health services in Mexico and in similar regions of resource-limited LMICS.

## 1 Introduction: pharmacogenomics and its relevance to public health systems

Pharmacogenomics (PGx) is the study of how the genome influences an individual’s response to drugs, integrating pharmacology, genetics, and genomic medicine. It analyzes how a person’s genetic nature affects their drug response, with the goal of optimizing pharmacotherapy based on the patient’s genotype to achieve maximum efficacy with minimal adverse effects (1–3). PGx involves the interaction of genetic variants—both germline and somatic—in drug-metabolizing enzymes, transporters, receptors, and other drug targets with pharmacokinetics (the Absorption, Distribution, Metabolism, and Excretion of drugs—ADME), pharmacodynamics (effects mediated by a drug’s biological targets), gene–gene and gene-food interactions, as well as immunogenic endpoints (4–9).

A large number of genes and multi-allelic genetic polymorphisms, which strongly depend on ethnicity, most notably those encoding the cytochrome P450 (CYP) superfamily of enzymes—responsible for the biotransformation of 70–80% of clinical drugs—and the *UGT* gene family, whose enzymes mediate critical detoxification reactions, are involved in this process (10–12). To date, more than 1,200 clinically relevant genetic variants linked to the ADME process have been identified (13). Furthermore, pharmacoepigenetic mechanisms and their variability are also beginning to gain importance in relation to drug response (14). While genetic analysis laboratories can design their own pharmacogenetic panels, they are typically based on the recommendations of the Clinical Pharmacogenetics Implementation Consortium (CPIC), ClinPGx (which includes PharmaGKB, CPIC, PharmCAT, ClinGen, and ClinVar), or the Dutch Pharmacogenetics Working Group (DPWG) (15–18).

A typical clinical panel can include between 15 and 100 genes related to pharmacokinetics and pharmacodynamics, depending on the platform and the clinical context (19). Each individual has different levels of enzymatic production derived from their genotype, and each drug is metabolized by different enzymes. Therefore, each person will have a distinct pharmacogenetic phenotype with respect to pharmacokinetic genes (colloquially referred to as metabolizer profiles). These are generally classified as: “normal metabolizers” (NMs), “poor metabolizers” (PMs, carrying two loss-of-function alleles), “intermediate metabolizers” (IMs, carrying one loss-of-function allele), and “ultrarapid metabolizers” (UMs, carrying gain-of-function alleles or gene duplications). For pharmacodynamic genes, designations such as positive or negative for high-risk alleles are used (16, 20).

The pharmacological scenario is even more intricate, some drugs are prodrug that require conversion into their active form, while others are already active and must be eliminated from the body (20). Depending on the metabolizer phenotype, a prodrug can be converted to its active form either very slowly or very quickly. The same applies to active drugs, which may accumulate or be eliminated more rapidly, altering their efficacy or toxicity (20, 21). Added to this complexity, some drugs also inhibit or induce the activity of the enzymes that metabolize them, indirectly affecting the pharmacokinetics of other co-administered medications, as well as those affected by certain food interactions (22). Decades of research into this interindividual enzymatic genetic variability have helped us understand why certain medications do not have the same effect on everyone or why they cause severe adverse reactions in some cases (23). Currently, it is estimated that ADRs account for approximately 7% of total hospitalizations in various countries (24). This figure can be considerably higher in older adults undergoing multiple treatments (polytherapy) (25, 26). However, reported ADR-related hospitalization rates vary widely across health systems and are influenced by underreporting, diagnostic coding practices, prescribing patterns, population characteristics, and the maturity of pharmacovigilance and health information systems. According to data from the Spanish health system, approximately one-third of diseases are not treated adequately due to pharmacological variability and therapeutic errors (27, 28).

This situation presents an opportunity for public health systems. The incorporation of pharmacogenetic analysis may allow for a potential optimization of the efficiency, safety, and cost-effectiveness of medical treatment. Increased therapeutic precision is associated with fewer hospitalizations, lower pharmaceutical costs, and a better clinical prognosis (29).

These examples are provided to illustrate the molecular basis underlying pharmacogenomic variability; however, the present work is framed within a public health systems and policy perspective, integrating concepts of health equity, systems innovation, and preventive ethics. Rather than presenting pharmacogenomics as an isolated technological intervention, the manuscript conceptualizes its implementation as a structural component of medication safety strategies, aimed at reducing preventable health harms and optimizing resource use at the population level.

## 2 The global landscape: lessons from early adopters

The application of pharmacogenomics at the primary care level within public health systems represents a timely need that has already begun to materialize in several countries, including both high-income countries and LMICs, through the implementation of precision medicine programs. While pharmacogenomics has not been their sole focus, it has been a major component. Among the most advanced pioneers are the United Kingdom, the United States, China, Spain, Greece and Brazil, all of whom have committed to structured precision medicine models based on the integration of genomics.

The United Kingdom has implemented several programs as part of its natural progression toward incorporating pharmacogenomics into public health services. These include the Centre of Excellence in Regulatory Science and Innovation in PGx (CERSI in PGx) to clarify often-confusing regulatory pathways; the PROGRESS study, a proof-of-concept for demonstrating IT models that support the integration of pharmacogenomics; the NHS England Genomic Test Directory, a central directory for genomic tests that clarifies what tests are offered and under what criteria; and finally, the Network of Excellence for Pharmacogenomics and Medicines Optimisation (PGx-NoE) in 2024, which has brought together key stakeholders from academia, health professionals, regulators, and civil society in two national meetings to define the next steps. These efforts have been driven in part by the positive results of the Pre-emptive Pharmacogenomic Testing for Preventing Adverse Drug Reactions (PREPARE) study within the European Ubiquitous Pharmacogenomics (U-PGx) project, which provided some evidence that the use of pharmacogenomic panel-based testing (12 genes and 44 variants) to guide prescribing can reduce the incidence of clinically relevant and potentially preventable adverse drug reactions associated with actionable drug-gene interactions within reported ranges of approximately 10–30%, depending on context and implementation (30–32). Although a pharmacogenomic model is not yet fully implemented in the UK as of 2025, these programs represent a coordinated national effort toward gradual, system-wide integration, making the UK one of the global frontrunners in pharmacogenomic implementation planning.

The United States took its first major step with the “Precision Medicine Initiative” launched in 2015 under the Obama administration, with an initial investment of $215 million for genomic research applied to the clinic. Of this, more than $130 million was funneled to the NIH, $70 million to the National Cancer Institute (NCI), $10 million to the FDA, and $5 million to the ONC (33, 34). This led to the creation of the “All of Us” program, which has the following updates as of today: The program’s genomic dataset has grown by nearly 70% to include whole genome sequences from more than 414,000 participants. Within this extensive collection, the program has identified more than 1.2 billion genetic variants, including more than 200 million previously unreported genetic variants. The number of people with Fitbit data has quadrupled to now include information from nearly 60,000 participants, creating the largest public dataset of wearable device information available, with some records extending back more than a decade. Enrollment in All of Us continues nationwide, currently with more than 849,000 participants across the United States and its territories. The Researcher Workbench currently powers more than 15,000 studies. In total, more than 15,000 researchers from all 50 states and more than 1,000 organizations worldwide are registered to use the platform (35).

For its part, China has led in absolute investment, committing more than USD 9.3 billion to its national precision medicine research and development plans under the 14th Five-Year Plan (2021–2025), a 43% increase from the previous five-year period. The Chinese Ministry of Science and Technology reports that the national Precision Medicine Initiative launched in 2016 had developed 22 national precision medicine research facilities by 2023, with annual funding topping ¥8.5 billion (USD 1.3 billion) (36).

In Spain, the Council of Ministers approved a call in 2020 to launch the Precision Medicine Infrastructure associated with Science and Technology (IMPaCT), the first step of the new Spanish Strategy for Personalized Medicine, financed by the Carlos III Health Institute. Its implementation and development were co-led by the Ministry of Health and the Ministry of Science and Innovation, with the participation of all autonomous communities. The project received an approximate investment of 77.3 million euros (25.8 million in grants and 51.5 million in 2021) (37). By 2025, the IMPaCT infrastructure is advancing across all of Spain, with 44 participating centers and implemented in all autonomous communities, seeking to follow 200,000 people for 20 years. It is worth noting that 70% of the participating centers are located in urban areas and the rest in rural areas (27, 38).

The case of Greece is very interesting because, despite having a significantly smaller population and an infinitely smaller budget than the other leading nations, it was the first country to launch a large-scale genome project in 2010 with GoGreece. This made it not only a pioneer in pushing for the equitable democratization of precision medicine, but also in designing implementation models for large-scale national genomic projects for resource-limited LMICs, such as the case of Brazil, where Greece’s model and implementation team played a key role (39). These countries have demonstrated that a successful implementation of precision medicine and PGx has enhanced personalized care and improved both clinical and financial outcomes (29, 40–42). These models serve not only as an inspiration but as clear evidence that the incorporation of genomics and pharmacogenomics into public health is not a futuristic concept, but a timely and feasible reality with proper planning, political will, and long-term vision.

While these international experiences provide valuable insights, their direct transferability to the Mexican public health context is inherently limited. Differences in health system structure, financing mechanisms, regulatory maturity, workforce capacity, health information infrastructure, and population-level prescribing patterns imply that observed clinical and economic outcomes—such as reductions in clinically relevant and potentially preventable adverse drug reactions reported in studies like PREPARE—are context-specific and intervention-dependent. For this reason, these examples are presented not as directly generalizable models, but as illustrative reference points to inform the design of a gradual, locally adapted pharmacogenomics implementation strategy.

## 3 The case for Mexico and Latin America

The situation in Mexico, and in Latin America more broadly, is complicated. Demographic, educational, cultural, and traditional factors, along with the predominant socioeconomic level—which, according to the latest official INEGI data (43, 44), places 56.1% of the Mexican population in the low socioeconomic stratum (44)—are compounded by poor nutrition and the excessive consumption of sugary beverages and ultra-processed foods (45, 46). This positions the country as one with the highest rates of type 2 diabetes, obesity, and cardiovascular diseases in the world (47–49). This is without even considering the complex genetic diversity generated by high levels of “mestizaje” (mixed-ancestry) in the Mexican population, together with the still limited and heterogeneous allele frequency variability data available for key pharmacogenes such as *CYP2D6*, where multiple Tier 1 and Tier 2 alleles show well-characterized frequencies in African, European, and Asian populations but remain sparsely characterized in Latino and Indigenous American groups (50). This gap represents both a scientific opportunity and a structural challenge for the design and implementation of population-level pharmacogenomic interventions. According to Salinas-Rodríguez et al. (51), polypharmacy in Mexico has reached levels of a global epidemic, as demonstrated in 45% of a cohort of older adults. While this reflects a substantial and growing pharmacological burden within the health system, it has not been paralleled by a proportional modernization of biomedical infrastructure or the systematic incorporation of pharmacogenomic strategies at the national level.

In this context, pharmacogenomics should not be understood as a standalone solution to systemic health system challenges, but rather as a potentially contributory tool that, when integrated with broader public health and regulatory strategies, may help mitigate avoidable pharmacological risks in selected populations. From a health equity standpoint, it is important to recognize that the uneven distribution of pharmacogenomic knowledge and infrastructure may risk widening existing gaps in medication safety. In Mexico and across Latin America, socially and economically vulnerable groups are often disproportionately exposed to chronic disease and polypharmacy. In this context, the absence of locally adapted pharmacogenomic strategies may contribute to preventable harm in specific settings. Framing PGx implementation through an equity-oriented lens emphasizes the importance of ensuring that the potential benefits of precision medicine extend beyond high-income settings or privileged subpopulations, including those with the greatest unmet needs (30, 39, 43, 44, 46).

In Mexico, one of the earliest organized efforts to promote pharmacogenomics was undertaken by researchers of the Academia de Genómica, CIIDIR-Durango, of the Instituto Politécnico Nacional of Durango, in collaboration with the Ibero-American Network of Pharmacogenetics and Pharmacogenomics (RIBEF), established in 2006. Over the past 17 years, this group has made important pioneering contributions, particularly by highlighting the role of ethnicity and genetic admixture in pharmacogenomic variability among Indigenous and admixed populations in Latin America (52–54). Their initiatives, including the MESTIFAR project, generated valuable population-based data and promoted awareness of the importance of genetic diversity in clinical pharmacology. While the overall clinical impact in Mexico has so far been modest, these sustained efforts deserve recognition as the first attempt to introduce pharmacogenomics into the national research agenda and to integrate Latin American diversity into the broader international conversation.

## 4 Barriers to implementation

The transition from theory to practice faces a dual challenge: immediate operational hurdles and deep-seated structural barriers. In the short term, the main obstacles involve administrative bandwidth, the readiness of the healthcare workforce, and acute resource gaps. Looking further ahead to the long term, the successful rollout of PGx depends on more complex, slow-moving processes—ranging from regulatory maturity and infrastructure development to sustainable financing and the large-scale educational shifts needed within medical institutions. At present, several regions report shortages of essential medications, gaps in primary care services, deteriorated facilities, and resource constraints affecting healthcare professionals. These limitations are not unique to Mexico and are frequently observed across resource-limited countries, yet they represent significant barriers to the adoption of PGx within the public health system. This creates a perception (unfortunately justified) that genomics and pharmacogenomics are a luxury reserved for high-specialty hospitals accessible only to a few.

Evidently, if the infrastructure for basic healthcare services remains insufficient, it becomes more challenging to establish the necessary components required to implement genetic and pharmacogenomic analyses. The technical barriers include:

- Insufficient molecular laboratories and sequencing equipment
- A deficit of trained technical and clinical personnel
- Fragmented processes
- The absence of fully interoperable electronic health records (EHRs), with current maturity remaining heterogeneous and institution-dependent due to multiple coexisting platforms and non-uniform data structures, although recent federal digital health reforms establish a legal framework for progressive EHRs interoperability (55)
- A shortage of medical geneticists (estimated at 1 for every 525,000 inhabitants in Mexico) (56)

Adding to this is a structural challenge in continuing medical education: if clinical staff remain unaware of, or unconvinced about, the importance of pharmacogenomics, they are less likely to request or prescribe it. Without this professional demand, institutional leaders are unlikely to prioritize it or generate the policy support required for its incorporation.

In parallel, Mexico’s General Health Law, Official Mexican Standards, regulations, and clinical guidelines must be updated. This process involves design, consultation, drafting, presenting initiatives, legislative discussion, promulgation, and official publication, a process that in any country can take years.

Finally, ethical concerns, particularly those related to informed consent, data privacy, and equitable access, should not be seen as obstacles but as structural pillars that must be addressed before any implementation effort begins. These are central aspects for ensuring that pharmacogenomics is a tool for equity and not for exclusion.

## 5 Opportunities and potential benefits

Among the potential system-level opportunities associated with the implementation of pharmacogenomics at public health institutions, the prevention of hospitalizations related to clinically relevant and potentially preventable ADRs can be pointed out as a critical opportunity for health public institutions. As obtained through the information provided by the National Center for Pharmacovigilance (CNFV) of COFEPRIS, ADRs account for approximately 7% of hospitalizations in Mexico (57).

In 2024, the Instituto Mexicano del Seguro Social (IMSS), the largest public health institution in Mexico, reported approximately 2.28 million hospital discharges (egresos hospitalarios) and ~10.56 million inpatient bed-days nationwide, according to its official financial and operational report to the Federal Executive and Congress (58). Applying an international benchmark suggesting that approximately 7% of hospitalizations may be attributable to ADRs, this would imply that on the order of ~159,000 ADR-associated hospital admissions per year could occur within IMSS alone.

To illustrate the economic implications of ADR-related hospitalizations, IMSS was used as a benchmark institution due to the availability of standardized, publicly reported unit costs per inpatient hospital day. Based on official cost schedules published in the Diario Oficial de la Federación (59) and reported inpatient bed-days (58), annual hospitalization expenditure within IMSS can be conservatively approximated at ~$158,000 million MXN. Applying the same illustrative benchmark fraction, ADR-associated hospitalization costs would be on the order of ~$11,100 million MXN annually within IMSS. An illustrative stepwise calculation underlying these estimates is presented in the following section.

These estimates should be interpreted with caution and understood as illustrative, hypothetical, contextual benchmarks. Reported ADR-related hospitalization rates vary substantially across health systems and are influenced by underreporting of ADRs, diagnostic coding practices, prescribing patterns, population-specific allele frequencies, variation in health IT maturity, and differences in gene-panel composition.

### 5.1 Illustrative pharmacogenomic impact

According to a review analyzing the cost-effectiveness of pharmacogenetic-guided treatment across pharmacogenomic (PGx) associations listed in the FDA Table of Pharmacogenomic Biomarkers in Drug Labeling, Verbelen et al. identified 44 published economic evaluations corresponding to 10 drugs among a total of 137 PGx associations. Of these economic evaluations, 57% concluded in favor of PGx-guided treatment compared with alternative strategies, including 30% classified as cost-effective and 27% as dominant (cost-saving). When the cost of genetic testing was hypothetically removed—reflecting scenarios in which genetic information is already available—75% of evaluations supported PGx-guided treatment, of which 25% were cost-effective and 50% were dominant. These findings assume that PGx-guided treatment may be not only cost-effective but, under certain conditions, cost-saving (29).

Illustrating a scenario-based hypothesis, a 10–30% reduction in preventable ADR-related hospitalizations within the IMSS benchmark context would correspond to illustrative savings of approximately $1,111–$3,333 million MXN annually from hospitalizations alone, without accounting for additional downstream savings related to reduced length of stay, lower use of unnecessary medications, medical litigation, and improved quality of life, as presented below.

Illustrative stepwise hypothesis (IMSS benchmark-based):

(i) Approximate annual inpatient activity within IMSS (2024): Based on its official financial and operational report to the Federal Executive and Congress, IMSS registers approximately 2.28 million hospital discharges per year, corresponding to a total of ~10.56 million inpatient bed-days annually (58).
(ii) Approximate ADR-attributable fraction of hospitalizations (international benchmark): Using cited international estimates (24, 57), ~7% of hospitalizations may be attributable to adverse drug reactions, corresponding to ~159,000 ADR-associated admissions per year within IMSS.
(iii) Approximate annual hospitalization expenditure within IMSS: Applying official unit costs per inpatient hospital day (15,018 MXN) published in the Diario Oficial de la Federación (59) to reported IMSS (58) inpatient bed-days (~10.56 million annually) yields an estimated annual hospitalization expenditure of ~$158,000 million MXN.
(iv) Approximate ADR-attributable hospitalization expenditure (benchmark-based): Applying the same illustrative 7% fraction (24, 57) suggests ~$11,100 million MXN annually in hospitalization costs potentially associated with ADRs within IMSS.
(v) Illustrative preventable fraction under panel-based pharmacogenomic implementation: Assuming a hypothetical 10–30% reduction in preventable ADR-related hospitalizations, consistent with results reported in panel-based PGx implementation studies (e.g., PREPARE) (30–32), this would correspond to illustrative savings of ~$1,111–$3,333 million MXN per year, from hospitalization costs alone.

These estimates are solely illustrative and are sensitive to assumptions regarding unit-cost definitions, the proportion of preventable ADRs mediated by actionable drug–gene interactions, population allele frequencies, pharmacogenomic panel composition, and real-world implementation fidelity. Additional details on parameter sensitivity are provided in the Supplementary Material.

In addition to the illustrative hypotheses presented here and the clinical outcomes reported by the U-PGx PREPARE initiative, peer-reviewed health economic evaluations conducted in low- and middle-income country settings, as well as within publicly funded European health systems—including economic analyses derived from the PREPARE study—have reported cost-effectiveness of pharmacogenomic-guided prescribing in public healthcare contexts (60–62).

Therefore, investing in pharmacogenomics may contribute to:

- Increased clinical effectiveness through more informed prescribing (right drug, right dose, right patient)
- A potential reduction in preventable adverse events and rehospitalizations
- Improved efficiency of pharmaceutical and hospital spending
- Progress toward greater equity in healthcare

Furthermore, such an approach may represent a step toward more modern, sustainable, and personalized public health systems, potentially aligning Mexico with precision medicine standards already adopted by other countries (39).

Beyond the illustrative calculations and estimates of preventable ADR-related hospitalizations and potential savings, the absence of pharmacogenomic integration can, in certain contexts, result in serious consequences at the individual level, as exemplified below in two illustrative clinical vignettes that underscore the potential value of its implementation.

### 5.2 Illustrative clinical vignettes in the absence of pharmacogenomic consideration

The absence of pharmacogenomic analysis in medical practice may contribute to preventable iatrogenic outcomes in certain contexts, particularly in high-risk clinical conditions where polypharmacy is common. The following two published clinical vignettes are included as illustrative sentinel examples to contextualize potential clinical consequences, rather than as population-level evidence.

As a first example, we present the case of 75 patients who underwent a kidney transplant and were medicated with tacrolimus and omeprazole. While 71 patients progressed positively, four of them showed a steady decline. Pharmacogenetic analysis revealed that the four patients who worsened were homozygous CYP2C19*2/*2 (rs4244285 AA). The study showed that the *1/*1 and *1/*2 genotypes, with normal metabolizing activity, allowed omeprazole to be metabolized by its preferred enzyme and tacrolimus by its own, *CYP3A5*. However, the *2/*2 genotype implied lower enzyme functionality, leading to an increase in unmetabolized omeprazole. This was associated with competitive inhibition of tacrolimus metabolism by *CYP3A5*, producing an increase in its blood levels and, consequently, allograft delayed function (acute tubular necrosis) in three of the patients. In this context, the lack of pharmacogenomic consideration may have contributed to serious clinical consequences (63).

In a second case, a 74-year-old-woman was admitted to the cardiology department with congestive heart failure, atrial fibrillation, and chronic renal failure. Several medications were prescribed to control the symptoms, which triggered erythrodermic psoriasis that was treated with oral and topical steroids, in turn causing neuropsychiatric symptoms that were aimed to be managed with more medications, accumulating a total of 18 drugs. Recommendation of pharmacogenomic analysis revealed *CYP3A4* oversaturation and relevant variants (homozygous for CYP3A5*3 and heterozygous for *MDR1* 3435C > T), explaining the ADRs. Instead of further intensifying steroids, physicians suspended, replaced and adjusted several drugs and doses based on her metabolizer profile, contributing to clinical stabilization (64).

These two illustrative clinical vignettes highlight the complexities inherent to empiric prescribing approaches in high-risk contexts.

As pharmacogenomic tools become more accessible, personalized prescription approaches may contribute to reducing the likelihood of certain preventable ADR incidents and related hospitalizations. The integration of pharmacogenomic considerations into clinical practice frameworks may also have implications for medico-legal risk management in complex prescribing environments, as standards of care evolve over time.

## 6 Toward a conceptual framework for pharmacogenomics implementation in public health

To integrate the pharmacogenomics (PGx) model into public health systems, particularly in low- and middle-income countries (LMICs), a conceptual and preliminary framework is proposed, based on interdependent building blocks that serve as core components for a gradual implementation: clinical pilots in high-morbidity settings, workforce and laboratory capacity, regulatory and ethical alignment, real-world data and evaluation, and public-private-academic partnerships. These interdependent building blocks are summarized in Table 1.

**Table 1:** Interdependent building blocks proposed for gradual implementation of PGx in public health.

Building block	Description/Rationale	Key considerations for Mexico and LMICs
Clinical pilots in high-morbidity populations	Prioritize cost-effective PGx panels focused on cardiovascular, metabolic, psychiatric, oncological, anti-infective, and analgesic drugs to demonstrate early clinical impact.	High prevalence of chronic diseases and polypharmacy offers immediate benefit and measurable outcomes.
Workforce and infrastructure	Train physicians, pharmacists, and lab personnel in PGx basics; establish local genotyping facilities and standardized workflows.	Severe shortage of medical geneticists (approximately 1 per 525,000 inhabitants); limited sequencing and bioinformatics infrastructure.
Regulation and ethics	Consider integration of PGx into clinical practice guidelines; ensure protocols for informed consent, equity, and access.	Align with national regulations; address disparities between high-specialty hospitals and primary care units.
Data collection and real-world evaluation	Implement interinstitutional EHRs, monitoring tools, pharmacovigilance, and cost-effectiveness analyses to generate real-world evidence, using prospective data to validate clinical outcomes.	Need for interoperable EHRs; fragmented health system complicates nationwide data collection.
Public-private-academic partnerships (PPAPs) as catalysts for implementation	Collaborations to provide genotyping platforms, training, and cloud-based bioinformatics.	PPAPs can bridge funding gaps; precedent set by Brazil’s DNABr initiative.

**Figure 1:**
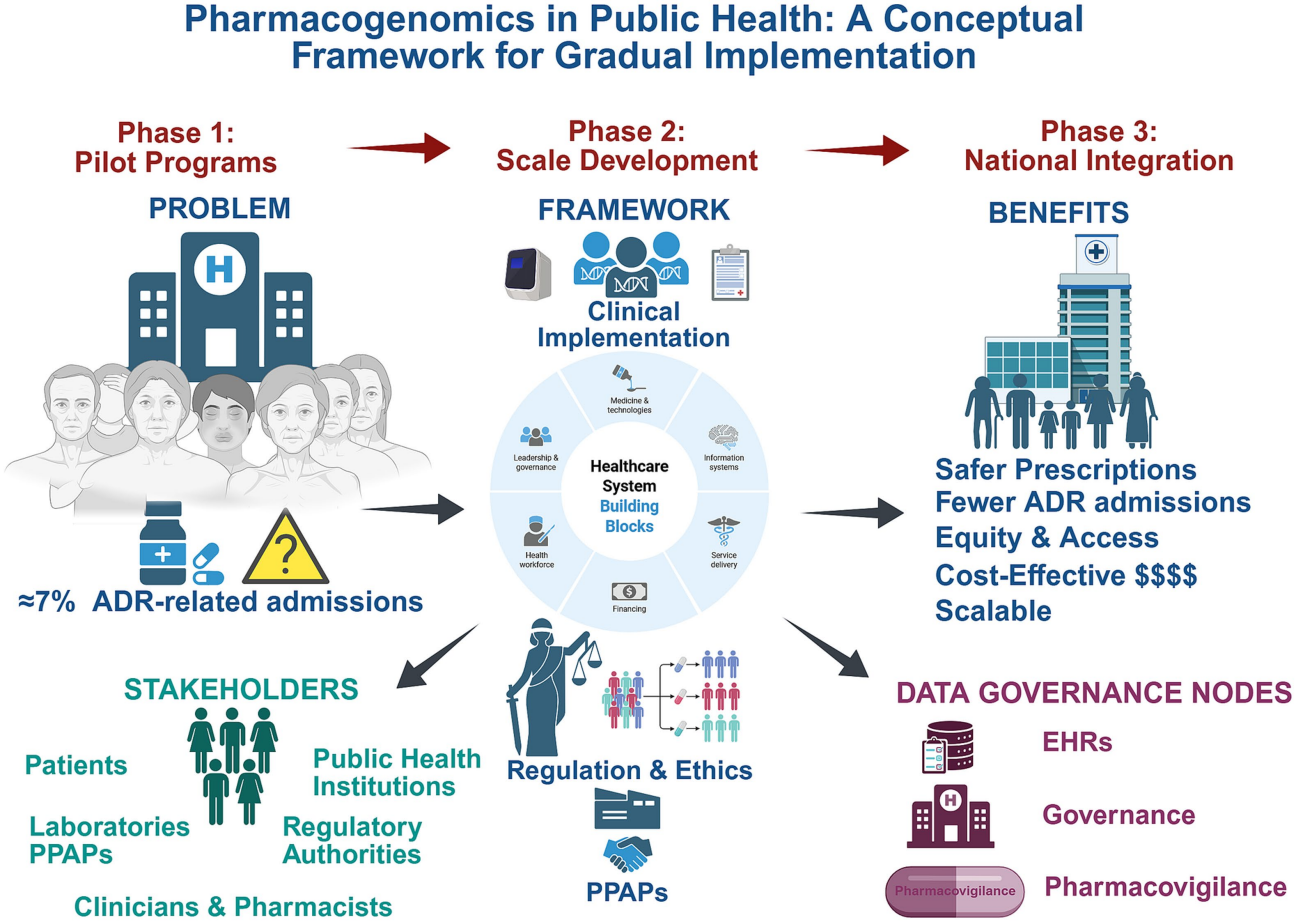
Conceptual framework for the gradual implementation of pharmacogenomics in public health systems, highlighting the key challenges, a phased implementation strategy (pilot, scale-up, and national integration), core health system building blocks, key stakeholder roles, and data governance nodes linking pharmacogenomic results with electronic health records and pharmacovigilance systems. The framework emphasizes regulatory oversight, ethical governance, and public–private–academic partnerships as enabling mechanisms for scalable and equitable adoption. Created in https://www.biorender.com/.

Figure 1 illustrates the conceptual framework for pharmacogenomics implementation in public health systems, highlighting the key challenges, a phased implementation pathway (pilot, scale-up, and national integration), the interdependent health system building blocks required for gradual deployment, and the governance and stakeholder components necessary for scalable implementation.

Based on international models, including the aforementioned UK’s PGx-NoE, the US’s All of Us, and primarily Spain’s IMPaCT and Greece’s Go-Greece—which has been described as a potential model for resource-limited countries—a conceptual plan can be proposed for Mexico and other Latin American countries. A key distinction compared to models implemented in highly resourced settings is the proposal to prioritize pharmacogenomics as an initial step, with gradual integration of additional precision medicine components over time.

Future empirical validation of this conceptual framework would rely on real-world implementation studies assessing process indicators (e.g., uptake, feasibility, and adherence), clinical outcomes (e.g., ADR reduction), and health-system metrics (e.g., hospitalization rates and cost-related indicators), within specific institutional and regulatory contexts.

### 6.1 Clinical pilots in high-morbidity settings

The initial implementation of a PGx program should prioritize regions and institutions that handle high levels of chronic degenerative diseases and polypharmacy. A viable, accessible, and low-cost pharmacogenomic panel should be designed, focusing on therapeutic groups with the strongest pharmacogenomic evidence and highest burden of morbidity, such as cardiovascular and metabolic, psychiatric and neurological, oncological, anti-infective, and analgesic medications. The goal is to guide clinical decisions and use previous analyses from native and mixed-ancestry populations as a benchmark to maximize impact while minimizing start-up costs (65, 66). This approach adheres to the five “R’s” for drug therapy: “the Right dose of the Right drug for the Right indication in the Right patient at the Right time,” and perhaps what has been suggested as a sixth R: “the Right ethnicity” (14).

Accordingly, an illustrative prioritization of specific drug–gene pairs—anchored in CPIC and DPWG guideline frameworks and contextualized to the Mexican public health setting—is presented in Table 2, based on the following criteria: (i) ADR severity and preventability; (ii) prescribing relevance in public institutions; (iii) availability of actionable guideline recommendations; and (iv) feasibility for integration into low-cost, panel-based testing within a stepwise implementation model. Tiering details are provided in the accompanying note. An extended version of Table 2 including key actionable haplotypes/alleles is presented in the Supplementary Material to ensure technical completeness while preserving clarity in the main text (9, 18, 30, 31, 58, 67–69).

**Table 2:** Illustrative prioritization of high-actionability PGx pairs for a Mexico-oriented public-sector pilot.

Therapeutic area	Drug	Gene(s)	Guideline source (CPIC/DPWG)	Guideline-supported actionability	Priority tier
Cardiovascular (antiplatelet)	Clopidogrel	*CYP2C19*	CPIC; DPWG (where applicable)	High (guideline-supported prescribing action)	Tier 1
Anticoagulation	Warfarin	*CYP2C9, VKORC1*	CPIC; DPWG (where applicable)	High (guideline-supported prescribing action)	Tier 1
Dyslipidemia	Simvastatin	*SLCO1B1*	CPIC; DPWG (where applicable)	High (guideline-supported prescribing action)	Tier 1
Analgesia	Tramadol	*CYP2D6*	CPIC; DPWG (where applicable)	High (guideline-supported prescribing action)	Tier 1
Psychiatry (SSRIs)	Escitalopram/Citalopram/Sertraline	*CYP2C19, CYP2D6*	CPIC; DPWG (where applicable)	High (guideline-supported prescribing action)	Tier 1
Oncology (fluoropyrimidines)	5-Fluorouracil/Capecitabine	*DPYD*	CPIC; DPWG (where applicable)	High (guideline-supported prescribing action)	Tier 1
Oncology (irinotecan)	Irinotecan	*UGT1A1*	CPIC; DPWG (where applicable)	High (guideline-supported prescribing action)	Tier 1
Oncology/Immunology	Azathioprine/6-Mercaptopurine/Thioguanine	*TPMT, NUDT15*	CPIC; DPWG (where applicable)	High (guideline-supported prescribing action)	Tier 1
Infectious diseases (TB)	Isoniazid	*NAT2*	CPIC; DPWG (where applicable)	High (guideline-supported prescribing action)	Tier 1
Transplantation (immunosuppression)	Tacrolimus	*CYP3A5*	CPIC; DPWG (where applicable)	High (guideline-supported prescribing action)	Tier 2
HIV	Abacavir	HLA-B*57:01	CPIC; DPWG (where applicable)	High (guideline-supported prescribing action)	Tier 2
Gout	Allopurinol	HLA-B*58:01	CPIC; DPWG (where applicable)	High (guideline-supported prescribing action)	Tier 2

(1) Guideline-supported actionability indicates the existence of established clinical pharmacogenomic guidelines—including those from the Clinical Pharmacogenetics Implementation Consortium (CPIC) and/or the Dutch Pharmacogenetics Working Group (DPWG)—that provide explicit, actionable prescribing recommendations, including drug selection, dose adjustment, alternative therapy, or contraindication, based on defined genotypes or inferred metabolizer phenotypes. (2) Tier classification reflects implementation priority within a public-sector pilot framework. Tier 1 drug–gene pairs were prioritized based on high clinical actionability, association with clinically significant and preventable adverse drug reactions, substantial prescribing relevance in public health institutions, and immediate feasibility for integration into low-cost, panel-based testing. Tier 2 pairs represent clinically actionable scenarios with lower population prevalence, more specialized clinical contexts, or reduced immediate impact at the population level, and are therefore proposed for subsequent implementation phases. This tiering approach is informed by established pharmacogenomic implementation frameworks and international public health deployment experiences.

### 6.2 Workforce and infrastructure

A critical barrier in LMICs is the lack of trained professionals and laboratory infrastructure. Training general practitioners or pharmacists and chemists in the basic principles of PGx and integrating this subject into curricula represents a critical component of implementation feasibility (70). Simultaneously, the establishment of genotyping laboratories and process standardization will be fundamental, along with the implementation of digital interfaces that can ensure scalability.

### 6.3 Regulation, clinical governance, and ethical oversight

Implementation of PGx in public health systems requires a distinct regulatory and governance framework to ensure analytical validity, clinical utility, patient safety, and ethical oversight. In Mexico, the primary regulatory authority overseeing the authorization and classification of the PGx tests is the Federal Commission for the Protection against Sanitary Risk, known as COFEPRIS. During an initial implementation phase, PGx assays in public institutions would likely be introduced through laboratory-based validated methodologies, comparable to internationally recognized laboratory-developed test (LDT) models, and operating under existing clinical laboratory regulatory frameworks, according to quality standards comparable to those applied internationally. This approach mirrors early implementation pathways adopted in other health systems and allows flexibility as regulatory frameworks mature. For this reason, PGx testing is not envisioned as mandatory but clinically indicated and limited to specific high-actionability drug-gene pairs, particularly in contexts associated with severe and preventable adverse drug reactions. Results would serve as clinical decision-support tools, which would inform drug selection, dosing, or avoidance—not as stand-alone diagnostic imperatives. Given their stable nature, PGx results would potentially reside within electronic health records (EHRs) and be leveraged longitudinally across multiple prescribing instances over time, thus optimizing clinical value and cost-effectiveness of PGx testing. Potential linkage to a system of pharmacovigilance could facilitate the use of PGx data in adverse drug reaction reporting and management strategies.

Operationally, PGx services in Mexico are currently implemented within the existing regulatory framework for clinical diagnostic services. In practice, this includes laboratories registered with COFEPRIS via its corresponding “Aviso de Funcionamiento,” either performing the full analytical workflow in-house or operating as sample collection and pre-analytical services with downstream analysis conducted by national or international reference laboratories. In all cases, compliance with applicable Mexican Official Standards (NOMs) governing sample collection, handling, transport, biosafety, and laboratory processes is required, independently of where genomic analysis is performed. International laboratory accreditations (e.g., ISO-based quality systems, CAP, CLIA) complement—but do not replace—the national regulatory framework (55, 71–73).

Importantly, the current regulatory setting with regards to genetic and genomic testing in Mexico is a dynamic one. In recent years, a comprehensive review of the overall regulatory setting for genetic testing in Mexico was conducted as a result of an initiative by a multi-stakeholder national expert group comprised of key players like Fundación Mexicana para la Salud (FUNSALUD) and Instituto Nacional de Medicina Genómica (INMEGEN) (74). Evidently, this effort, which has also seen the participation of key players in the academic field, federal government, and both professional and patient groups, underscores a clear imperative with regards to updating and streamlining burgeoning regulations with regards to genetic technologies, which include DNA, RNA, and biomarkers for testing. Although these efforts have not resulted in published binding regulations, it is an important start that marks the natural transition from the current situation towards the relevance of the implementation of policy-oriented models such as the one proposed in this manuscript.

In the context of health institutions, reimbursement represents structural support and financing schemes rather than direct payment. In the current context, there are no specific pathways in place for the reimbursement of pharmacogenomics (PGx) tests in any health institution in Mexico, nor are these tests categorized with billing codes for such diagnostic activities. Therefore, once carried out, these tests are likely to be classified merely as a part of other diagnostic activity, research, or innovation schemes (75).

From a policy viewpoint, the lack of clear reimbursement mechanisms can be both a limitation and a future opportunity for PGx implementation. Instead of being immediately accommodated and integrated in the reimbursement pathways, PGx implementation may follow an incremental approach to be incorporated in specific programs for certain drugs, certain populations, or even as part of the diagnostic pathway in high-risk populations. This can help to generate local evidence on feasibility, clinical utility, and budgetary implications prior to broader coverage decisions.

As can be seen from international experience, pharmacogenomic reimbursement depends heavily on the context, including administrative, regulatory, and incentives structures, even in healthcare systems with established clinical guidelines and coding systems. There are significant reimbursement variations, as has been seen through real-world evidence from high-income countries. This re-emphasizes that reimbursement challenges often reflect structural and administrative factors in addition to evidentiary considerations. Under that paradigm, in early implementation phases, PGx testing may function primarily as a clinical decision-support tool rather than as a standalone reimbursable therapeutic service. The value proposition lies in its ability to inform prescribing decisions, decrease unnecessary adverse drug reactions, and ultimately optimize pharmaceutical utilization on a population basis, rather functioning as an isolated billable service. The same considerations would be especially pertinent to public sector funding models, where coverage decisions are intimately linked to demonstrating benefits at the population level and expenditure control priorities (76–78).

From a governance perspective, the model proposed aligns with prevailing international regulatory and ethical standards set by the International Council for Harmonisation (ICH) and the World Health Organization (WHO). Regarding financing mechanisms, it would be expected to be dynamic and to evolve progressively, starting with pilot programs in high impact areas, in order to inform subsequent policy decisions regarding a broader public sector adoption (79–82).

Integrating pharmacogenomics (PGx) into routine care brings up vital questions about where professional responsibility lies. In the model we propose, PGx testing is strictly a clinical decision-support tool; it is neither prescriptive nor a replacement for medical judgment. Consequently, the final word on any treatment remains with the physician, a principle that aligns with long-standing medical practice standards. It is crucial to clarify that using PGx data does not automatically shift liability to laboratories or digital platforms. Instead, accountability is shared across the clinical chain: from the lab generating the data to the specialist interpreting it, and finally, the physician applying it to the patient’s specific context. To keep this process legally sound and safe, institutions must establish clear protocols that define these roles explicitly. From a systemic viewpoint, PGx cannot exist in isolation. Its success depends on solid governance, including standardized reports, updated clinical guidelines, and access to specialized support for complex cases. These mechanisms do more than just improve quality; they reduce medico-legal risks by framing PGx as one more piece of the clinical puzzle—an informational input—rather than an isolated mandate. In public health systems, where decisions are tied to institutional frameworks, these structures are essential. They ensure that technological innovation stays aligned with professional accountability, patient safety, and the ethical demands of public service (83–88).

In parallel with regulatory and operational considerations, the incorporation of pharmacogenomics into clinical practice guidelines requires strict alignment with existing legislation and health system norms. A critical component of this model is the close monitoring of ethical safeguards, including informed consent, data protection, and equitable access, to prevent the unintended amplification of health inequities (89–91).

### 6.4 Data collection and real-world evaluation

Building upon the regulatory and governance framework described above, the implementation of the PGx model may also include the integration of interinstitutional electronic health records (EHRs) to ensure data interoperability, complemented by data monitoring tools for clinical and operational oversight, cost analysis mechanisms to explore potential benefits, and pharmacovigilance. It would also be of great benefit to include the prospective collection of clinical data through genomic data access committees (DACs), to validate the clinical results of its use (92).

A gradual, region-focused implementation strategy—supported by evidence from pioneering countries—can help ensure that the model’s adaptation is cost-effective and equitable within Latin American public health infrastructures.

### 6.5 Integration of pharmacogenomics into ADR reporting and risk management

Pharmacogenomic findings could be incrementally added to existing pharmacovigilance and ADR reporting systems. Genotype-informed data have the potential to inform causality assessment by putting into context individual susceptibility to ADRs, notably in cases with known high-risk drug-gene interactions. In this setting, genotype-informed ADR data may also contribute to signal detection activities by contextualizing emerging safety signals according to known drug–gene risk profiles. Long-term linkage of pharmacogenomic findings to ADR reporting and EHRs would potentially allow for finer risk stratification, contribute to institutional risk management plans, and inform revisions to system-level prescribing guidelines and medication safety strategies (93–98).

### 6.6 Public-private-academic partnerships (PPAPs) as catalysts for implementation

In low-budget settings, and especially in the initial stages, public–private–academic partnerships (PPAPs) can play a strategic role in bridging existing gaps in infrastructure and technology. A compelling example is Brazil, where a private sector-sponsored WGS sequencing of 3,000 participants and the setup of the DNABr database enabled the launch of the DNABr project in December 2019. Shortly thereafter, the Ministry of Health provided funding for an additional 3,000 WGS and cloud computing (39). Establishing collaborations with private laboratories, biotechnology groups, and academic institutions could accelerate access to every aspect the model requires, including genotyping platforms, trained personnel, bioinformatics, and training programs. Efficiently regulated PPAPs may help ensure favorable cost–benefit balance, data integrity, and sustainability, while preserving equity as a fundamental principle in the implementation of pharmacogenomics services (39, 89, 99).

## 7 Conclusion: toward a pharmacogenomics-ready public health system

Pharmacogenomics represents an increasingly feasible and evidence-informed clinical technology for public health systems aiming to provide modern and equitable care. While its implementation in low- and middle-income countries (LMICs) presents logistical, operational, and financial challenges, the long-term benefits in terms of safety and cost-effectiveness have been documented in various health-system contexts. Conversely, the continued absence of pharmacogenomic integration may be associated with ongoing costs related to preventable ADRs, suboptimal treatment responses, and inefficient or misaligned resource utilization.

We consider the integration of pharmacogenomics into public health systems to be ethically and economically justified under current evidence and public health principles. This effort could begin in the areas most affected by the non-use of pharmacogenomics and progressively scale up to the entire system.

Future empirical research should evaluate pilot implementations of this framework in high-morbidity settings, assessing feasibility, clinical impact (e.g., reduction in preventable ADR-related hospitalizations), and health-system outcomes (e.g., resource utilization and cost-related indicators). Public-private-academic partnerships may represent a pragmatic mechanism for enabling such pilot programs and facilitating scalable implementation pathways. Evidence generated from these initiatives would allow refinement of implementation models and provide context-specific data to inform broader scale-up strategies.

Ultimately, pharmacogenomics may represent a progressively integrated component of public health systems seeking to enhance treatment precision and equity in access to care.

## Data Availability

The original contributions presented in the study are included in the article/Supplementary material, further inquiries can be directed to the corresponding author.
